# Supplementary material for: An evolutionary path to altered cofactor specificity in a metalloenzyme
Source: Nat Commun. 2020 Jun 1;11:2738. doi: 10.1038/s41467-020-16478-0 (PMC7264356; doi:10.1038/s41467-020-16478-0)
Supplement: Supplementary file 1 — Supplementary Information [file 41467_2020_16478_MOESM1_ESM.pdf]

SUPPLEMENTARY INFORMATION

An evolutionary path to altered cofactor specificity in a metalloenzyme

Anna Barwinska-Sendra[1], Yuritzi M. Garcia[2], Kacper M. Sendra[1], Arnaud Baslé[1], Eilidh S. Mackenzie[1], Emma Tarrant[1], Patrick Card[1], Leandro C. Tabares[3], Cédric Bicep[1], Sun Un[3], Thomas E. Kehl-Fie[2,4]*, Kevin J. Waldron[1]*

[1]Institute for Cell and Molecular Biosciences, Faculty of Medical Sciences, Newcastle University, Newcastle upon Tyne, NE2 4HH, United Kingdom.
[2]Department of Microbiology, University of Illinois Urbana-Champaign, Urbana, IL, 61801, USA.
[3]Department of Biochemistry, Biophysics and Structural Biology, Université Paris-Saclay, CEA, CNRS, Institute for Integrative Biology of the Cell (I2BC), 91198, Gif-sur-Yvette, France.
[4]Carl R Woese Institute for Genomic Biology, University of Illinois Urbana-Champaign, Urbana, IL, 61801, USA.

*These authors jointly supervised this work.

Authors to whom correspondence should be addressed:
Thomas E. Kehl-Fie, Phone: +1 217 244-5471, email: kehlfie@illinois.edu.
Kevin J. Waldron, Phone: +44 191 208, email: kevin.waldron@ncl.ac.uk.

Contents:
Supplementary Figures 1-12.
Supplementary Tables 1-7.
Supplementary Notes 1-2.
Supplementary References.

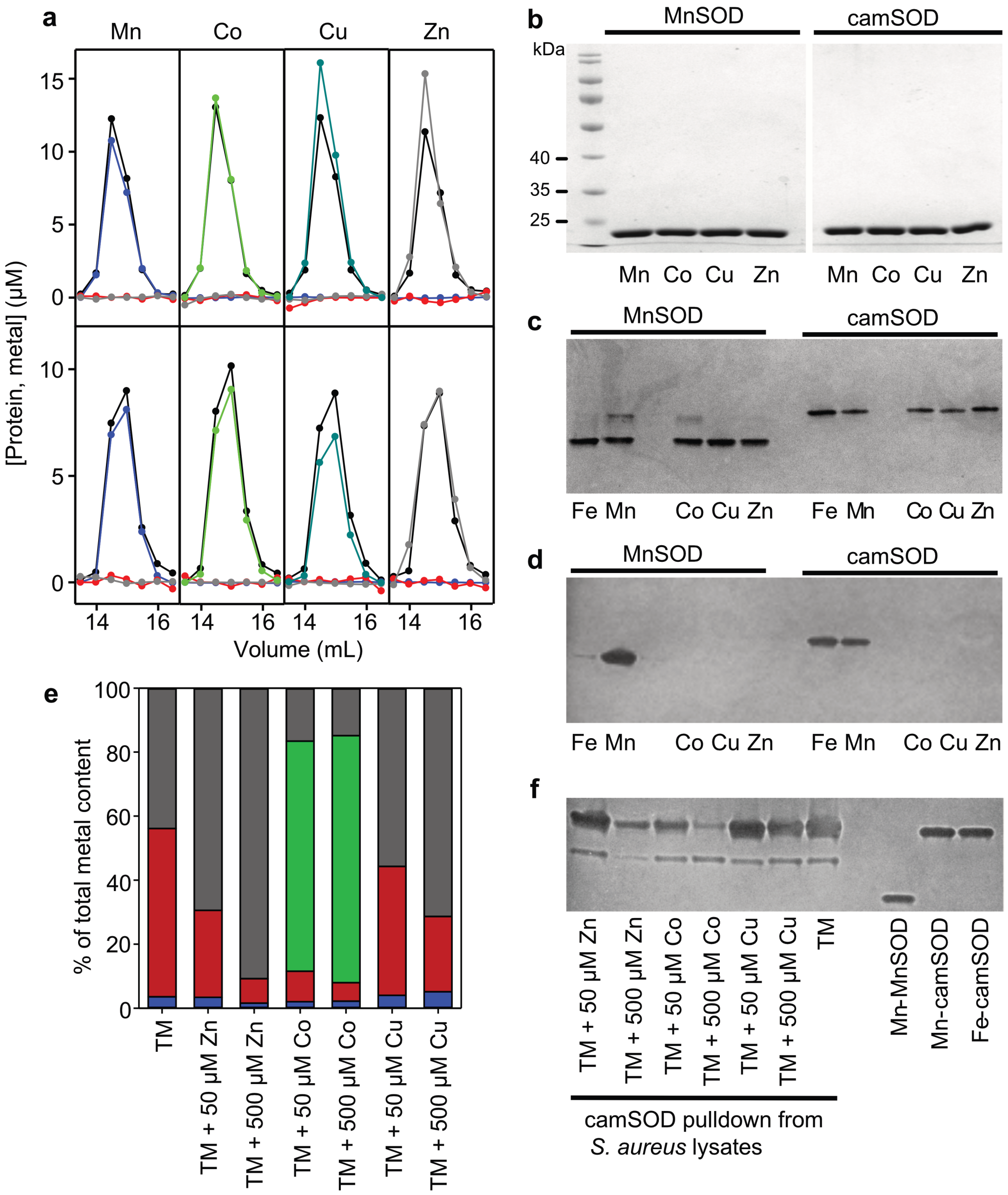

Supplementary Figure 1: Both *S. aureus* SODs are catalytically inactive when loaded with non-native metal ions *in vitro* and *in vivo*.

(a) Purified, recombinant wild type (upper panels) MnSOD or (lower panels) camSOD enzymes (black) were each loaded with manganese, or with the non-native metals cobalt, copper or zinc *in vitro*, by refolding of guanidine-denatured protein through dialysis in the presence of 1 mM of the relevant divalent metal ion[1] (*n*=1). After refolding, samples were resolved by size exclusion chromatography (Superdex 200 Increase, GE Healthcare) and analysed for protein content by A_{280nm}, using a correction factor derived from quantitative amino acid analysis[2], and for manganese (blue), iron (red), cobalt (green), copper (teal) and zinc (grey) by ICP-MS. All protein samples contained stoichiometric metal. (b-d) Analysis by (b) SDS-PAGE, with molecular weight (MW) markers (kDa) shown, and (c) native PAGE, each with Coomassie staining, as well as (d) in-gel SOD activity by nitroblue tetrazolium assay demonstrated that neither enzyme exhibits catalytic activity with any of the non-native metal cofactors. Note that MW markers were not resolved on native gels for Coomassie or in-gel activity stain, with the identity of each SOD band deduced instead by loading of recombinant enzyme controls. (e-f) Supplementation of *S. aureus* Tris minimal medium[3] (*n*=1) with low (50 µM) or high (500 µM) concentrations of these metals led to incorporation of cobalt or zinc (but not copper) in camSOD, as determined after pull-down of C-terminally Strep-tagged protein followed by ICP-MS metal analysis (e), with colours as described for panel (a), leading to a loss of camSOD activity detected in the resulting *S. aureus* lysates (f). Note that the kinetic trapping of the metal ion by the enzymes, and the instability of the apo-forms of the proteins have thus far precluded experimental affinity measurements. Source data for panels b-f are provided as a Source Data file.

```
Sa-MnSOD  MAFELPKLPYAFDALEPHFDKETMEIHHDRHHNTYVTKLNAAVEGTDLESKSIEEIVANL   60
Ss-SodA   MAFELPKLPYAFDALEPHFDKETMEIHHDRHHNTYVTKLNAAVEGTDLESKSIEEIVANL   60
Sg-SodA   MAFELPKLPYAFDALEPHFDKETMEIHHDRHHNTYVTKLNAAVEGTDLESKSIEEIVANL   60
----------------------------------------------------------------------------
Sa-CamSOD MAFKLPNLPYAYDALEPYIDQRTMEFHHDKHHNTYVTKLNATVEGTELEHQSLADMIANL   60
Ss-SodM   MAFKLPNLPYAYDALEPYIDQRTMEFHHDKHHNTYVTKLNATVEGTDLEHQSLADMIANL   60
Sg-SodM   MAFKLPNLPYAYDALEPYIDQRTMEFHHDKHHNTYVTKLNATVEGTDLEHQSLADMIANL   60
          ***:**:****:*****::*:.***:***:***********:****:**  :*:  :::***

Sa-MnSOD  DSVPANIQTAVRNNGGGHLNHSLFWELLSPNSEEKGTVVEKIKEQWGSLEEFKKEFADKA  120
Ss-SodA   DSVPANIQTAVRNNGGGHLNHSLFWELLSPNSEEKGTVVEKIKEQWGSLDEFKKEFADKA  120
Sg-SodA   DSVPADIQTAVRNNGGGHLNHSLFWELLSPNSEEKGTVVEKIKEQWGSLDEFKKEFADKA  120
----------------------------------------------------------------------------
Sa-CamSOD DKVPEAMRMSVRNNGGGHFNHSLFWEILSPNSEEKGGVIDDIKAQWGTLDEFKNEFANKA  120
Ss-SodM   DKVPEAMRMSVRNNGGGHFNHSLFWEILSPNSEEKGGVIDDIKAQWGTLDEFKNEFANKA  120
Sg-SodM   DKVPEAMRMSVRNNGGGHFNHSLFWEILSPNSEEKGGVIDDIKAQWGTLDEFKTEFANKA  120
          *.**  ::  :********:*******:********* *::.**  ***:*:***.***:**

Sa-MnSOD  AARFGSGWAWLVVNNGQLEIVTTPNQDNPLTEGKTPILGLDVWEHAYYLKYQNKRPDYIG  180
Ss-SodA   AARFGSGWAWLVVNNGQLEIVTTPNQDNPLTEGKTPILGLDVWEHAYYLKYQNKRPDYIG  180
Sg-SodA   AARFGSGWAWLVVNNGQLEIVTTPNQDNPLTEGKTPILGLDVWEHAYYLKYQNKRPDYIG  180
----------------------------------------------------------------------------
Sa-CamSOD TTLFGSGWTWLVVNDGKLEIVTTPNQDNPLTEGKTPILLFDVWEHAYYLKYQNKRPDYMT  180
Ss-SodM   TTLFGSGWTWLVVNDGKLEIVTTPNQDNPLTEGKTPILLFDVWEHAYYLKYQNKRPDYMT  180
Sg-SodM   TTLFGSGWTWLVVNNGKLEIVTTPNQDNPLTEGKTPILLFDVWEHAYYLKYQNKRPDYMS  180
          ::  *****:*****:*:******************** :*****************:

Sa-MnSOD  AFWNVVNWEKVDELYNATK   199
Ss-SodA   AFWNVVNWEKVDELYNAAK   199
Sg-SodA   AFWNVVNWEKVDELYNATK   199
-------------------------------------
Sa-CamSOD AFWNIVNWKKVDELYQAAK   199
Ss-SodM   AFWNIVNWKKVDELYQAAK   199
Sg-SodM   AFWNIVNWEKVDELYQAAK   199
          ****:***:******:*:*
```

Supplementary Figure 2: Sequence similarity of the pair of superoxide dismutase isozymes from *S. aureus*, *S. schweitzeri* and *S. argenteus*.
Amino acid sequence alignment of the SOD enzymes (MnSOD = SodA, camSOD = SodM) encoded in the genomes of a selection of staphylococci, including *S. aureus* (denoted *Sa*). The conserved amino acid residues that coordinate the metal cofactor are highlighted (red), as are all sequence positions that vary between the *S. aureus* MnSOD and camSOD (blue) isozymes, and the residues that were targeted for mutagenesis in this study (green). Sequence positions that show variation between the SOD enzymes in *S. aureus* and their counterparts in the genomes of the closely related species *S. schweitzeri* (denoted *Ss*) or *S. argenteus* (denoted *Sg*) are also highlighted (yellow). The alignment was produced using Clustal Omega[4]. The asterisk indicates fully conserved sequence positions, whilst the colons and semicolons indicate strongly and weakly similar sequence positions respectively.

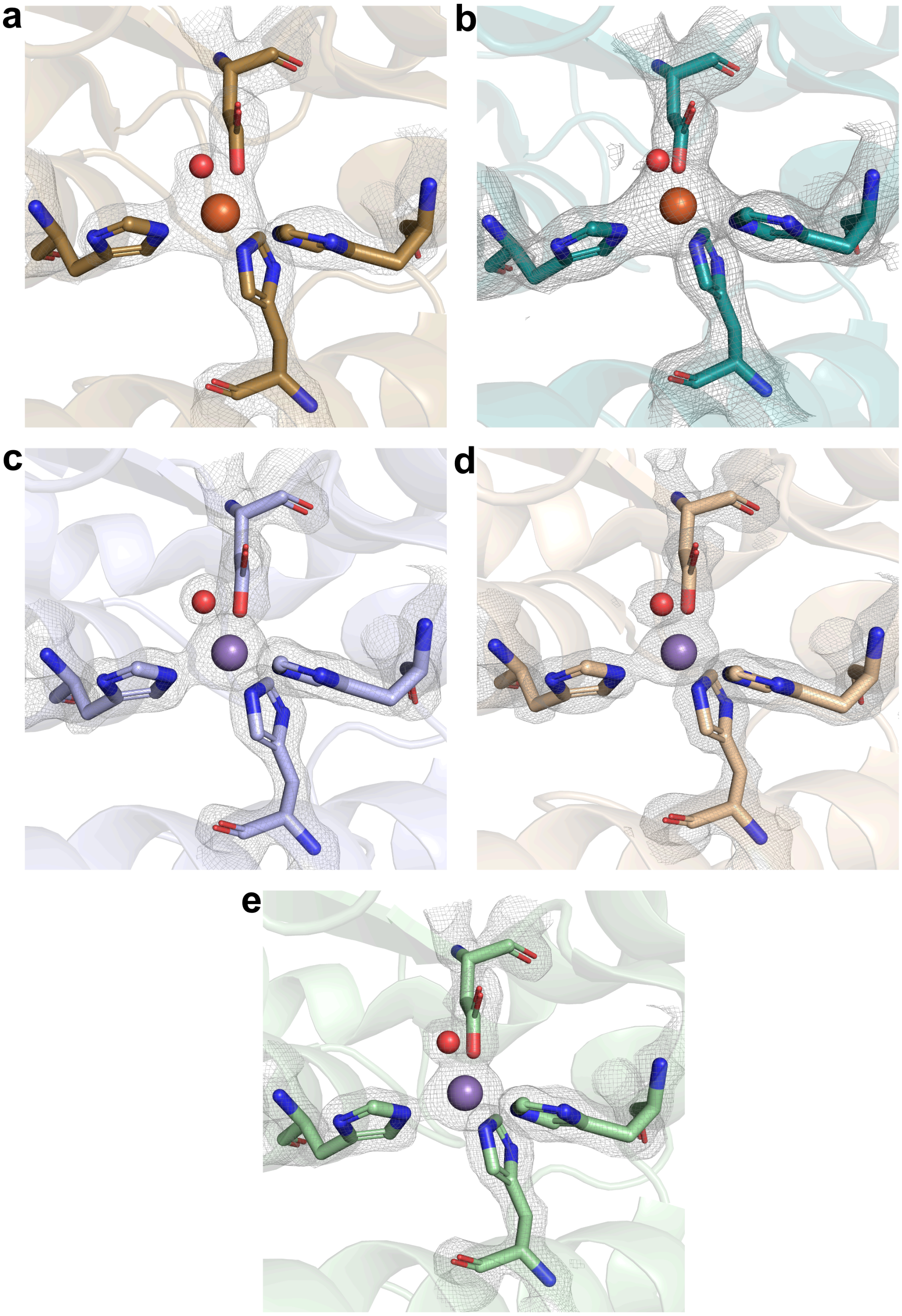

Supplementary Figure 3: Active site of SOD variants with electron density map.
Stick representation of the active site ligands of (a) Fe-MnSOD (gold), (b) Fe-camSOD (teal), (c) MnSOD (G159L-L160F) (light blue), (d) camSOD (L159G-F160L) (wheat) and (e) camSOD (I19F-L159G-F160L) (light green). Electron density map 2Fc-Fo represented as grey mesh, at 2 σ contour level in all presented structures, apart from (b) where the map is displayed at 1.5 σ contour level.

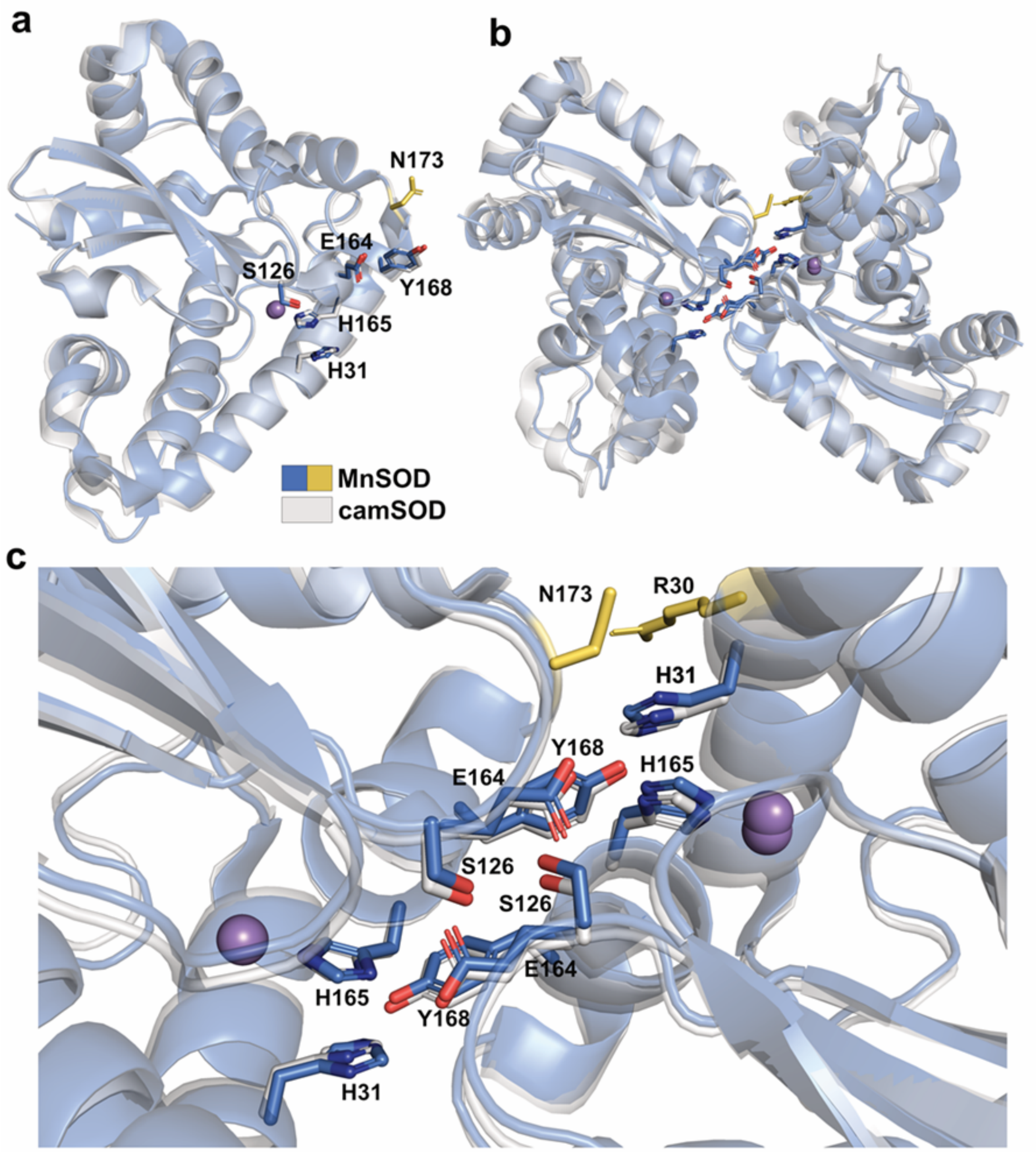

Supplementary Figure 4: The dimerization interface of the two *S. aureus* SODs are highly conserved.
Cartoon diagram showing overlaid (a) monomer and (b) dimer structures, and (c) a zoomed view of the dimerization interface, of MnSOD (blue ribbon) and camSOD (grey ribbon), with Mn(II) cofactors shown as purple spheres. Amino acid residues that line the dimerization interface in camSOD are shown in stick representation (grey), and are conserved in MnSOD (blue), which also possesses a further pair of residues at this interface (yellow) that are involved in this interaction. Of these, residue Asn173 is conserved in camSOD, whereas the MnSOD residue Arg30 is a lysine in camSOD (as well as in both *E. coli* enzymes), as shown in Supplementary Fig. 2. The identification of dimerization residues used PDBePISA[5] and images were produced in PyMol[6].

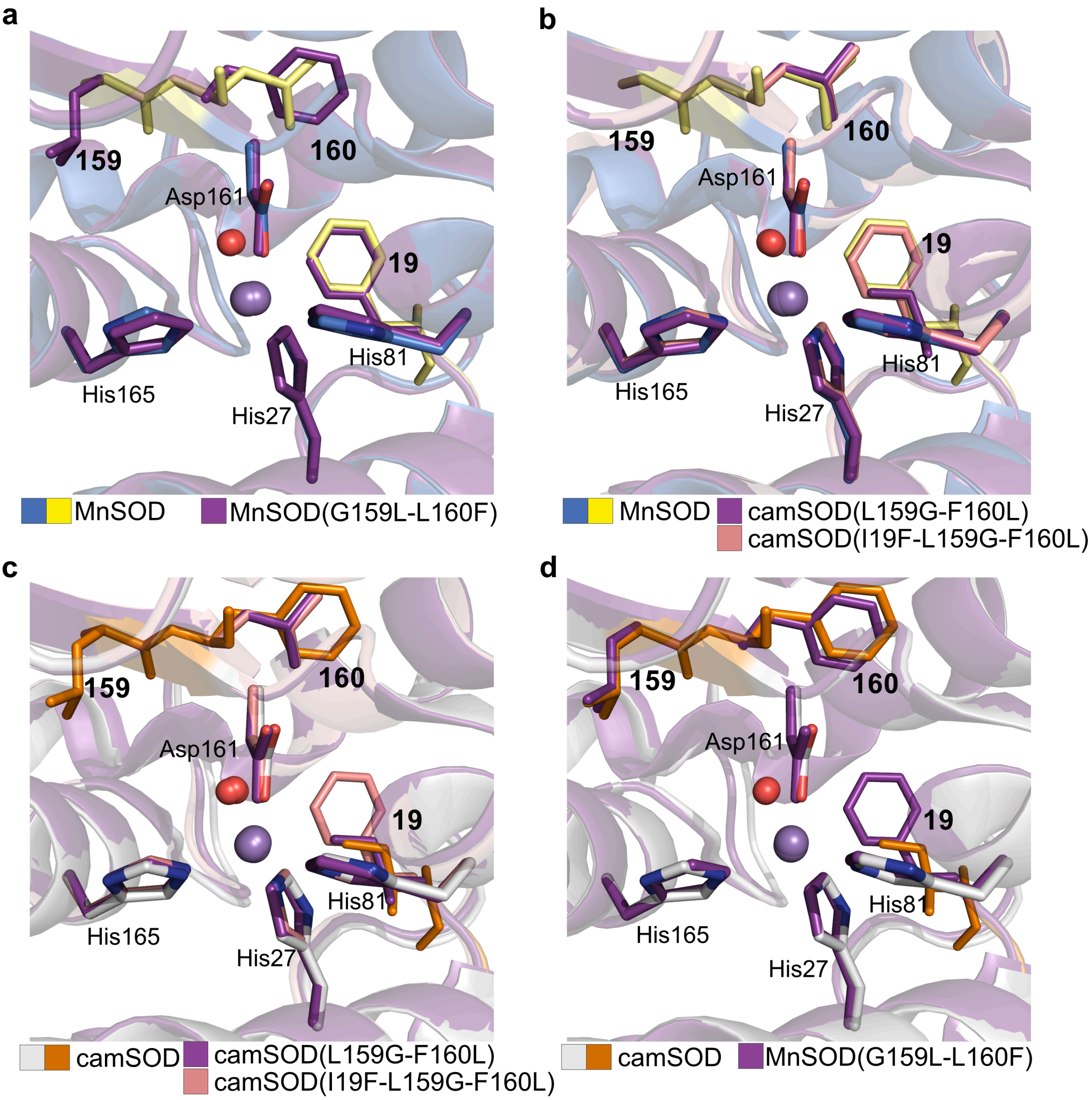

Supplementary Figure 5: Active site architectures of the mutated variant SOD enzymes are unchanged from wild type.
Cartoon representations demonstrating the similar active site architecture in wild type and mutant SOD crystal structures[2]. All structures shown were determined from crystals produced from manganese-loaded forms of the enzymes. (a) Structure of wild type MnSOD (ribbon and metal ligands in blue, mutated residues in yellow) overlaid with the double mutant variant MnSOD Gly159Leu-Leu160Phe (purple). (b) Structure of wild type MnSOD (colored as in panel a) overlaid with both the double mutant variant camSOD Leu159Gly-Phe160Leu (purple) and the triple mutant variant camSOD Ile19Phe-Leu159Gly-Phe160Leu (salmon). (c) Structure of wild type camSOD (ribbon and metal ligands in grey, mutated residues in orange) overlaid with the double mutant variant camSOD Leu159Gly-Phe160Leu (purple) and the triple mutant variant camSOD Ile19Phe-Leu159Gly-Phe160Leu (salmon). (d) Structure of wild type camSOD (colored as in panel c) overlaid with the double mutant variant MnSOD Gly159Leu-Leu160Phe (purple). The positions of the backbones were identical in the mutants within the limits of the structural resolution, and the positions adopted by the mutated sidechains were comparable to those of the corresponding sidechains in the wild type enzymes. Figures were prepared in PyMol[6]. For crystallization and structural determination statistics see Supplementary Table 3.

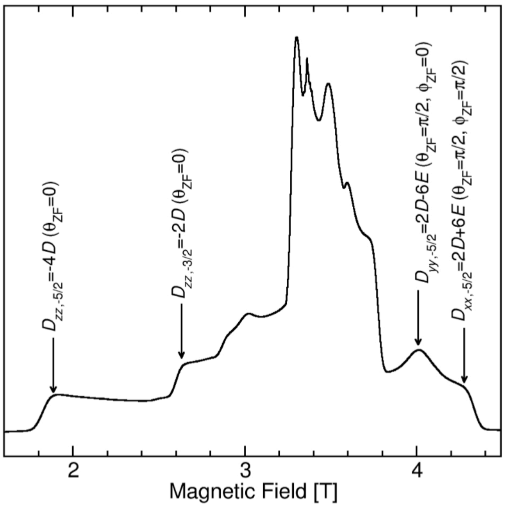

Supplementary Figure 6: Determination of *D* and *E* values from HFEPR spectra.
The zero-field *D* and *E* values were obtained directly from the spectra (Fig. 3a) using the three $D_{nn,-5/2}$ (*nn*=*xx, yy* and *zz*) field positions illustrated, as described in Supplementary Note 1.

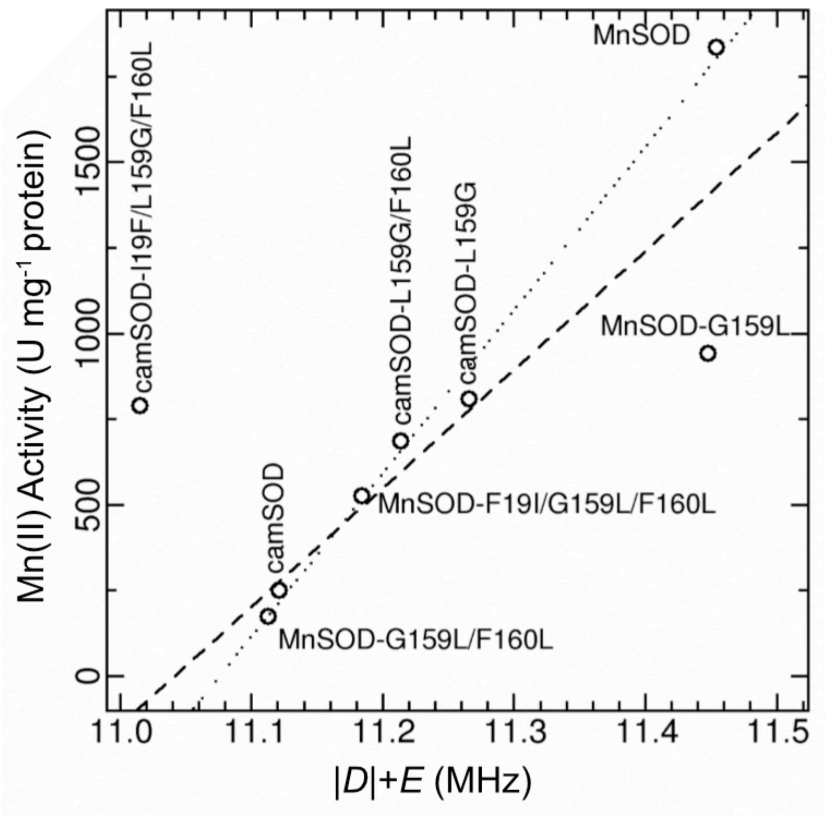

Supplementary Figure 7: Correlation between the zero-field interaction and Mn-dependent enzymatic activity of the Mn-loaded forms of the *S. aureus* wild type and variant SODs. High field electron paramagnetic resonance (HFEPR) spectroscopy was used to investigate how the magnetic zero-field interaction (ZFI) was affected by the mutations in the *S. aureus* SOD isozymes (Fig. 3a). The ZFI interaction is characterized by its *D* and *E* values, which are sensitive to the metal's electronic environment well beyond the immediate ligand sphere. We have previously shown that the magnitude of $|D|+E$ values can be used to distinguish the three different types of Mn/Fe SODs[7], including the wild-type *S. aureus* SODs[2]: MnSODs have the largest values, manganese-substituted FeSODs have the smallest, while those of cambialistic SODs are intermediate[7]. In all cases, the mutations had significant effects on the electronic environment of their Mn(II) centers. The $|D|+E$ values of the wild type and variant SODs followed their Mn-dependent activity, with the most significant deviation arising from camSOD Leu159Gly-Phe160Leu. The dashed line shows a linear regression fit that excludes this variant (r= 0.886) and the dotted line also excludes MnSOD Gly159Leu (r=0.996). All mutations had a pronounced effect on the electronic environment of the Mn(II) centers, even though they had no measurable effect on the protein structure by X-ray crystallography (Supplementary Fig. 4). It is important to note that our HFEPR measurements only reflect the reduced state and is insensitive to how Mn(III) centers are affected by the mutations. Information about how these parameters were calculated is presented in Supplementary Fig. 6 and Supplementary Note 1.

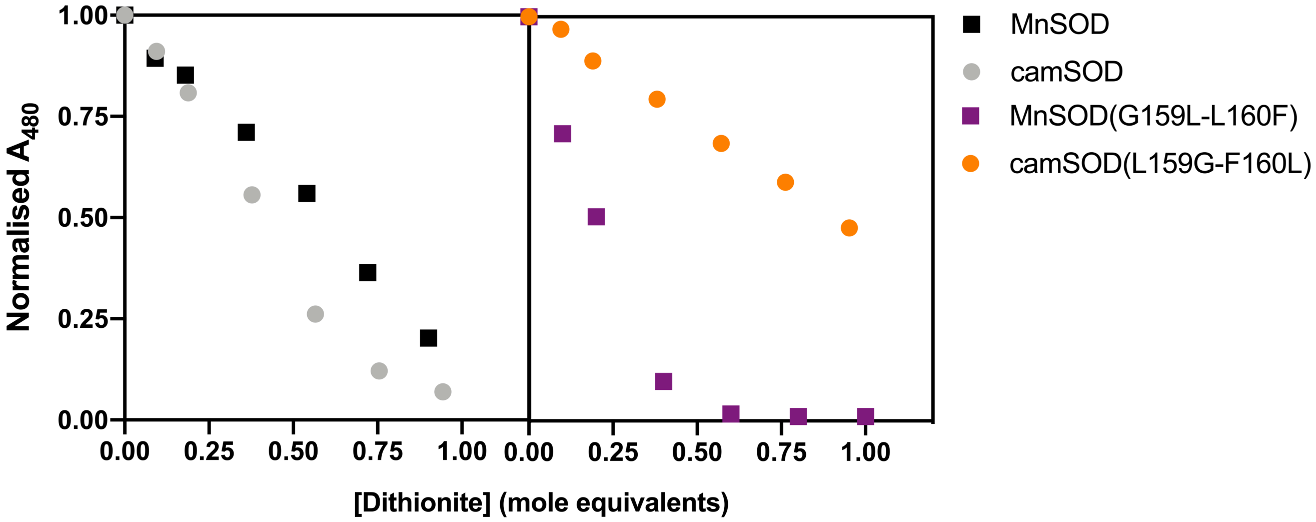

Supplementary Figure 8: The SODs exhibit differential susceptibility to dithionite reduction. To qualitatively test whether the *S. aureus* SODs differed in their reductive properties, each manganese loaded enzyme of (left) the wild type forms or (right) the double mutant variants was titrated with the reductant dithionite and the abundance of the oxidized, Mn(III) form of the enzyme was monitored at 480 nm by UV/visible spectroscopy (see Fig. 3). Each titration experiment was performed by repeated additions to a single protein sample in 100 mM phosphate buffer, pH 7.5, containing 100 mM KCl, 1 mM EDTA at room temperature in 1 cm pathlength quartz cuvettes. Absorbance values were corrected for protein concentration (~800 μM) adjusted to account for differences in manganese-loading and then normalised to allow direct comparison between the forms despite small differences between their spectra. Titration data demonstrated that the wild type camSOD (grey circles) became reduced on incubation with lower concentrations of reductant than MnSOD (black squares), whereas this trend was reversed in the mutants, with the MnSOD G159L-L160F variant (purple squares) becoming reduced at lower concentrations of reductant than the camSOD L159G-F160L variant (orange circles). The stability of the enzyme was tested before and after oxidation with permanganate, and after the dithionite titration by in-gel and quantitative spectrophotometric liquid activity assay, which demonstrated that full activity of all four forms was detected after both the oxidation and the reduction step. These data are from one biological replicate that was representative of further independent analyses (*n*=3).

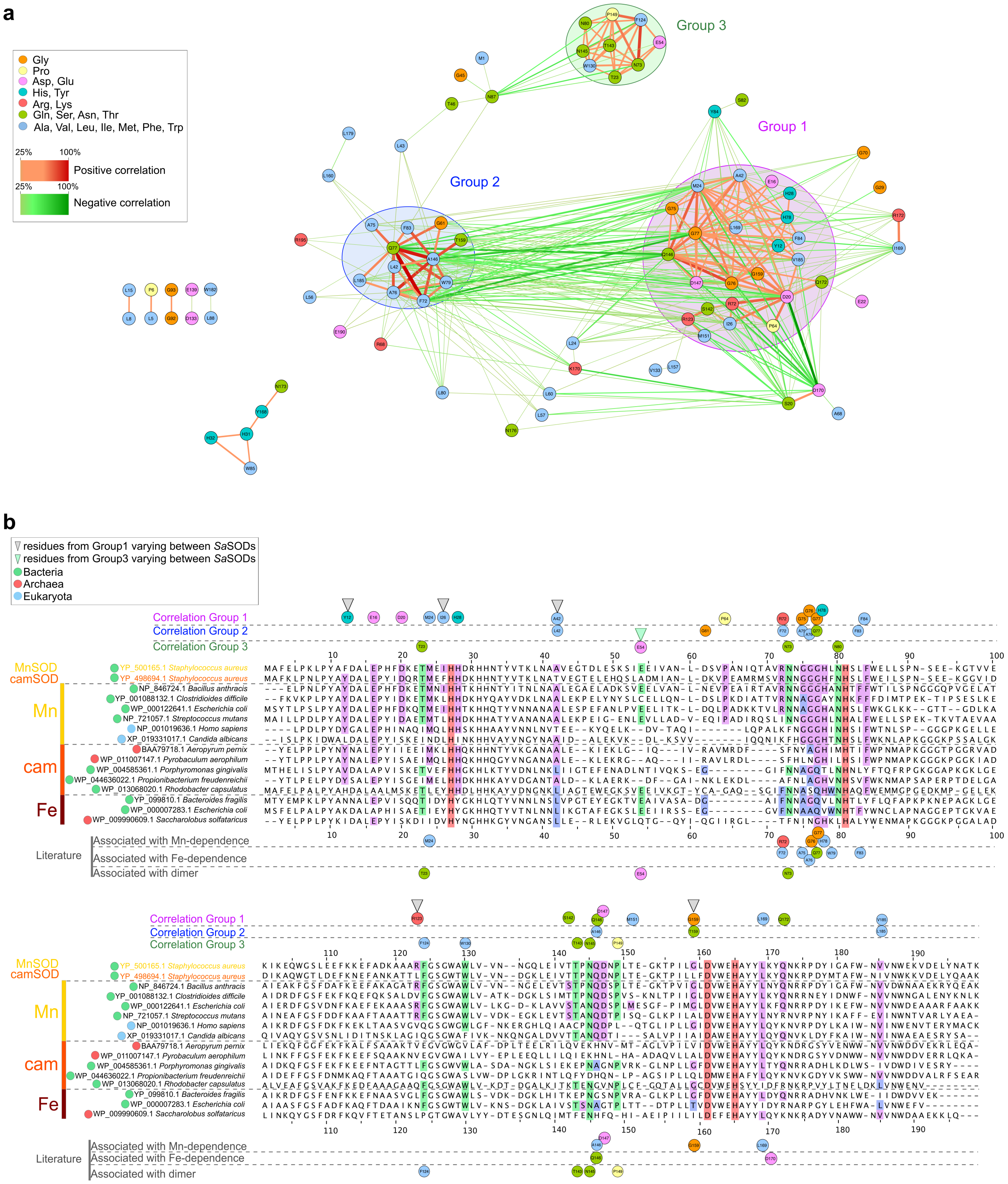

Supplementary Figure 9: Amino acid correlation network analysis of SOD sequences.
(a) Network representation of groups of co-evolving amino acids identified in the alignment of the 2,691 SOD sequences sampled across the tree of life (Supplementary Data 1) using amino acid correlation analyses (PFstats[9]). The circles (nodes) represent amino acid residues, and lines (edges) correspond to the pairwise positive correlation (red) or anti-correlation (green) between two nodes. The intensity and thickness of the edges correspond to the correlation value, where the highest absolute values were annotated as thickest with the most intense colour. Correlation values correspond to percentage of the maximum correlation scores, and minimum anti-correlation scores (Supplementary Data 2). Groups 1 (GGH: purple), 2 (AAQ: blue), and 3 (green) represent the groups of connected nodes containing more than 5 positively correlated residues.
(b) Full sequence alignment of a selection of SOD sequences of empirically confirmed metal specificity, shown in partial form in Fig. 4b, illustrating the positions of the metal binding residues in

red. Metal specificities (MnSOD: yellow; FeSOD: brown; camSOD: orange) of these characterized isozymes are illustrated (left), and their origins from archaea (red), bacteria (green), or eukaryotes (blue) are illustrated with *circles* next to the species name. Residues identified in correlation groups 1, 2, and 3 (colored as in panel a), as well as the conserved amino acid residues of functional and/or structural relevance described in the literature[10], were mapped onto the alignment. The alignment was extracted from the full alignment of 2,691 SOD homologues (Supplementary Data 1). Amino acid residue numbering corresponds to the *S. aureus* SOD sequences. The amino acid network was analyzed and presented using Cytoscape[11], and the alignment was visualized in JalView[12].

The sub-groups identified by the combined amino acid correlation and phylogenetic analysis is a reflection of the broad diversity and widespread occurrence of SODs in nature. The GGH correlation group contains confirmed primarily manganese-dependent SODs[13-15], but also contains enzymes shown to be both iron-dependent[16] and cambialistic[17-21]; the AAQ group contains primarily iron-dependent SODs[22,23], but also contains confirmed cambialistic enzymes[18,21]; the third group possesses neither conserved motif. Note that for the purpose of defining these metal specificities from the existing literature, rigorous cut-offs ($CR < 0.1$ = MnSOD; $CR > 0.1$ = camSOD; $CR > 10$ = FeSOD) were used to ensure only those of precisely defined metal specificity were included in the analysis (see Fig. 4a).

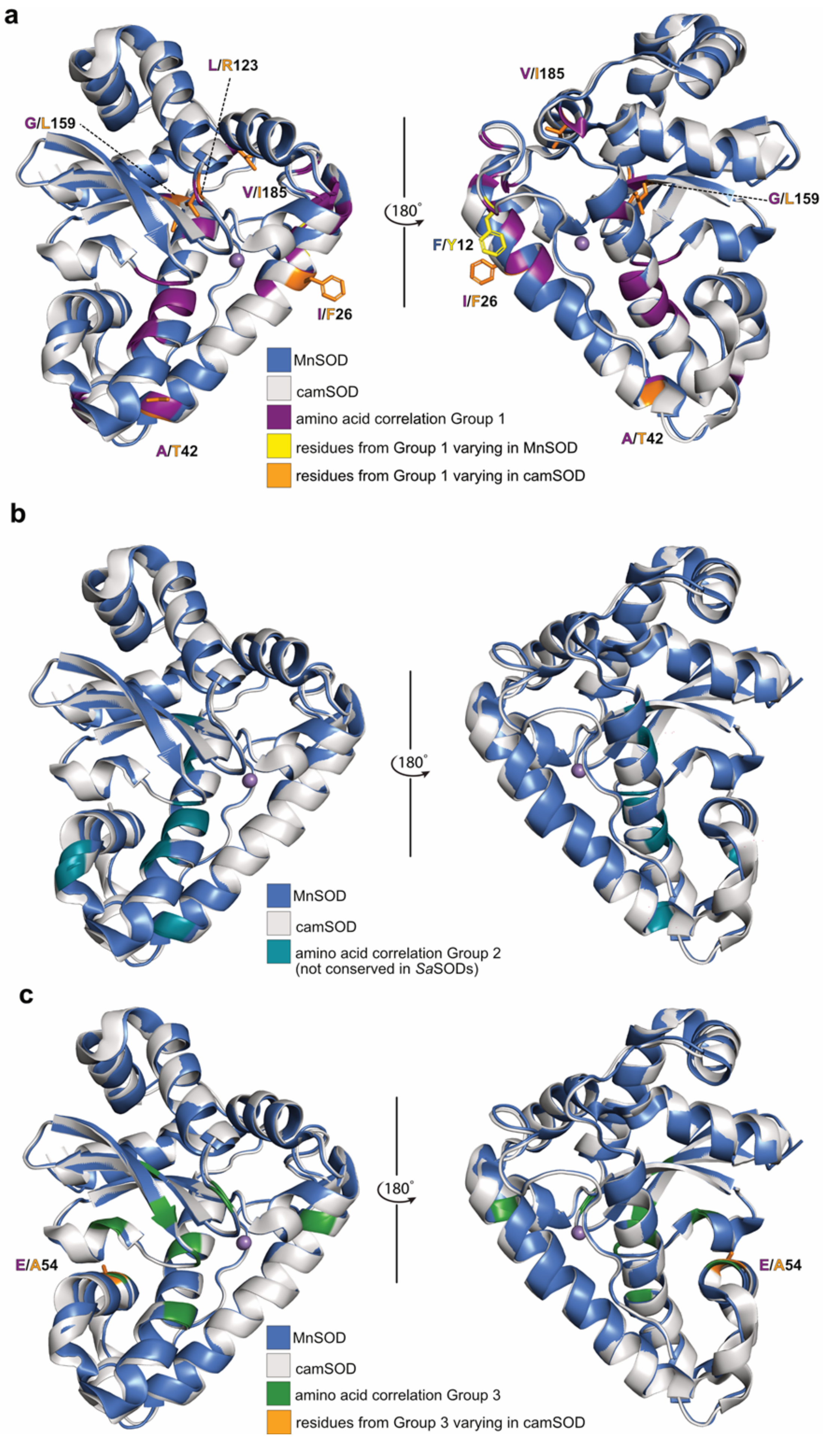
a
L/R123
G/L159
V/I185
I/F26
A/T42
180°
V/I185
G/L159
F/Y12
I/F26
A/T42
MnSOD
camSOD
amino acid correlation Group 1
residues from Group 1 varying in MnSOD
residues from Group 1 varying in camSOD
b
180°
MnSOD
camSOD
amino acid correlation Group 2
(not conserved in *Sa*SODs)
c
E/A54
180°
E/A54
MnSOD
camSOD
amino acid correlation Group 3
residues from Group 3 varying in camSOD

Supplementary Figure 10 (on previous page): Spatial localization of residues of the identified amino acid correlation groups within the SOD protein structure.

Overlaid polypeptide backbone cartoon representations of the crystal structures of MnSOD (blue ribbon) and camSOD (grey ribbon), with Mn(II) centers shown as purple spheres. (a) The structural positions of the residues identified by amino acid correlation analysis as defining the GGH sub-group are shown in purple on the ribbons, except for those GGH-correlated positions that are not conserved in MnSOD (yellow) or camSOD (orange) (shown as triangles above the sequence alignment in Supplementary Fig. 9). (b) The structural positions of the residues identified by amino acid correlation analysis as defining the AAQ sub-group are shown in green on the ribbons, none of which are conserved in either of the *S. aureus* SODs. (c) The structural positions of the residues identified by amino acid correlation analysis as defining the third sub-group (lacking both the GGH and AAQ motifs) are shown in cyan on the ribbons, except for those that are not conserved in camSOD (orange). Structural images were generated in PyMol[6].

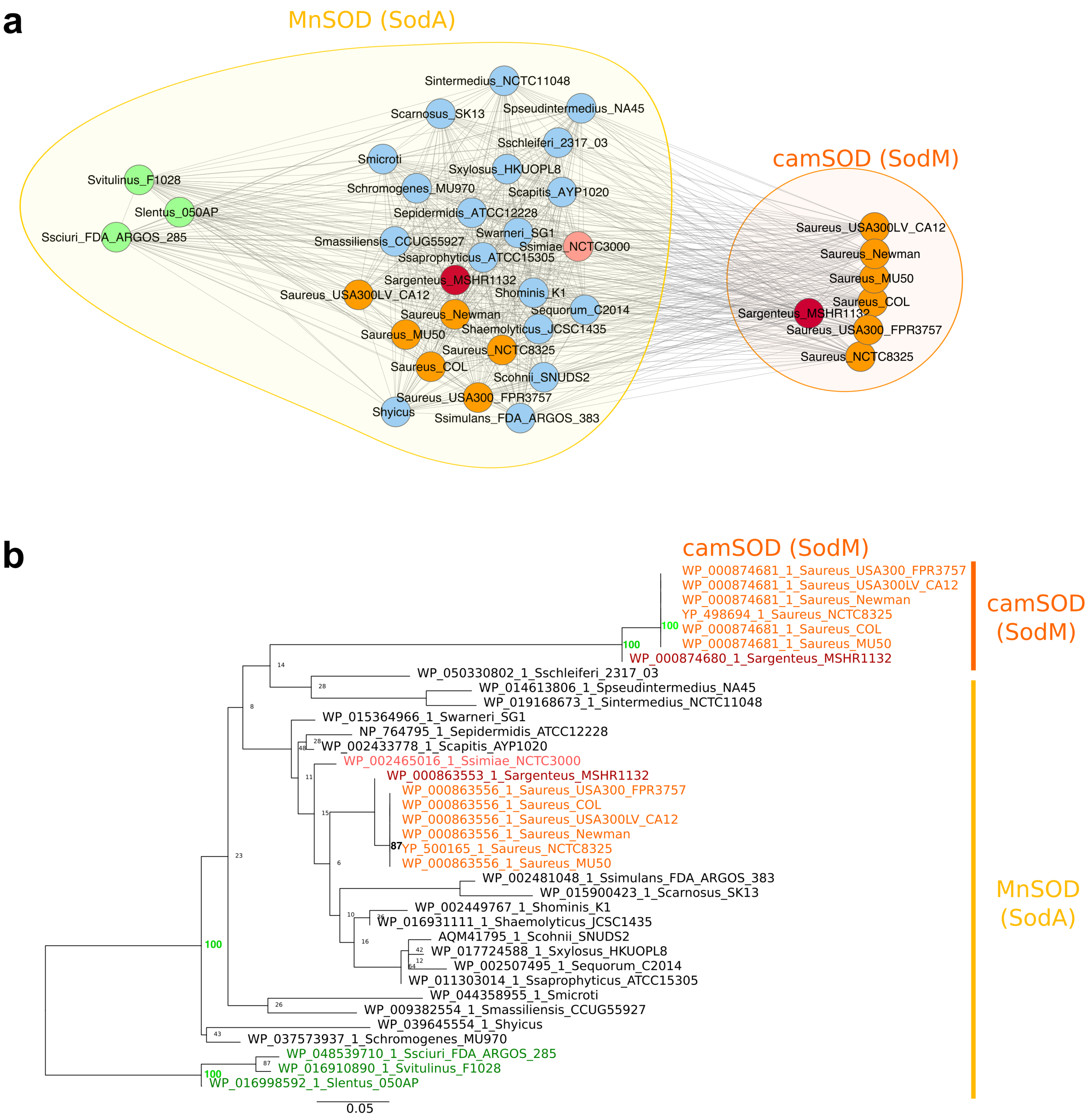

Supplementary Figure 11: Staphylococcal SOD network and phylogenetic tree.
(a) SOD network from the protein similarity network analysis of the 29 staphylococci (Fig. 5c), and (b) the phylogenetic tree constructed from the SODs identified in the network, illustrating the region of the network containing the staphylococcal SOD enzymes. The SOD network was initially identified as belonging to the Enriched in *S. aureus* networks, due to the high level of sequence identity (75% identical) between the camSOD and MnSOD isozymes. Subsequently, camSOD (SodM) and MnSOD (SodA) orthologues were identified and reclassified as *S. aureus*-specific, and Present in all, respectively, based on the phylogenetics (Fig. 4a, and illustrated here in panel b), protein similarity network topology (panel a), protein sequence analyses (Fig. 4b, Supplementary Fig. 9b), and whole genome analyses (Fig. 5a). The collection of MnSODs are grouped in yellow, and the camSODs are grouped in orange. Each node in (a) represents a single staphylococcal SOD sequence, and vertices represent pairwise sequence identity, with colors illustrating representatives from *S. aureus* (orange), *S. argenteus* (red), *S. simiae* (pink), other oxidase-negative staphylococci (blue in panel a, and black in panel b), and the outgroup containing oxidase-positive *S. sciuri*, *S. lentus*, and *S. vitulinus* in green. The maximum-likelihood phylogeny was generated under WAG+G model in RAxML with 100 rapid bootstrap replicates. Scale bar represents the number of substitutions per site, bootstrap support values above 90 were indicated in green.

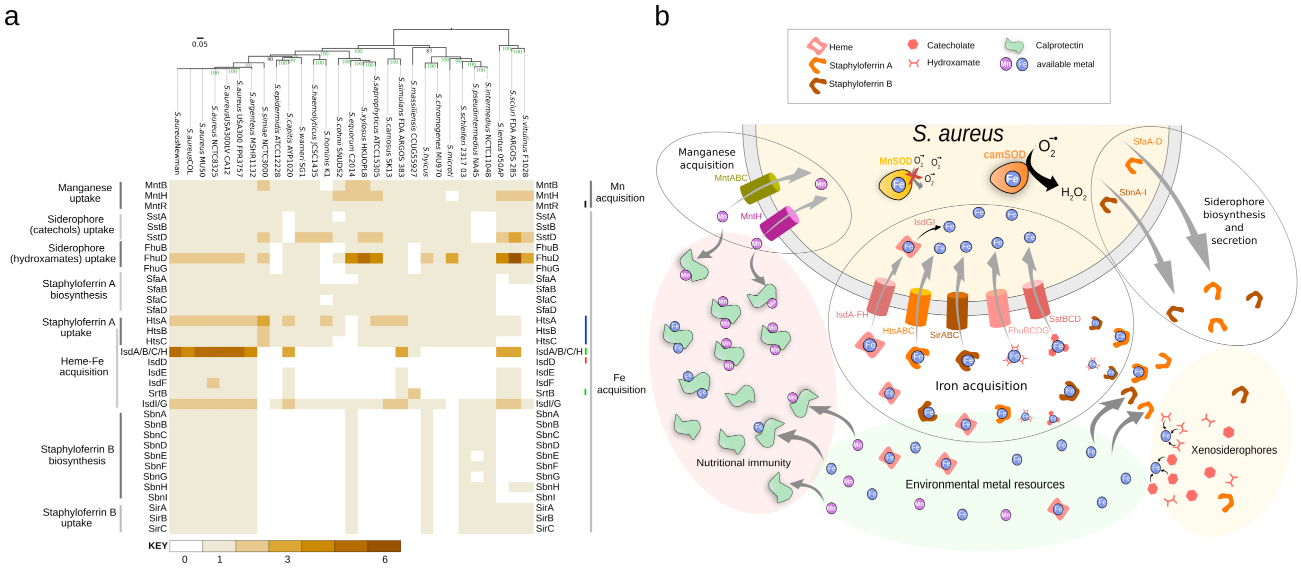

Supplementary Figure 12: Heatmap illustrating the distribution of known metal acquisition systems for manganese and iron across the staphylococci.
(a) Heatmap and (b) schematic diagram illustrating the distribution of selected proteins known to play a role in acquisition of manganese[24-27] and iron[28-30] detected in the network analysis (Supplementary Data 6). The *S. aureus*/*S. argenteus* lineage acquired or expanded its copy number of a number of proteins belonging to iron uptake systems, including via siderophore biosynthesis/uptake and via heme uptake, suggesting the ancestor of the *S. aureus*/*S. argenteus* lineage encountered changing iron availability and/or demand. All of the protein homologues displayed in the heatmap in panel (b) with the exception of the MntR (black, Identified in all), and the IsdD (red*, S. aureus*-specific), belonged to large networks of more complex protein families, and were identified based on the phylogenetic analyses. Essential genes are indicated in blue and virulence factors are indicated in green. For detailed definition of categories see online methods. The key represents the number of identified homologues.

Supplementary Table 1: Secondary structure content of recombinant forms of each variant of *S. aureus* MnSOD and camSOD.
A comparison of the secondary structure content (calculated percentage content of α-helix, β-sheet and other), as calculated from the X-ray crystallographic structural models (see Fig. 1b) using PDB-SUM[31] (labelled X-ray), with that calculated (*n*=1) from the empirical far-UV CD spectroscopy data (see Fig. 1a for example), obtained using Dichroweb[32] (labelled CD).

			Secondary structure (%)		
**Protein**	**Metal**	**Data**	**α-helix**	**β-sheet**	**Other**
**MnSOD**	**Fe**	X-ray	54.8	11.8	33.3
		CD	52.0	13.0	35.0
**MnSOD**	**Mn**	X-ray	52.6	11.6	35.9
		CD	52.0	12.0	36.0
**camSOD**	**Fe**	X-ray	57.1	11.6	31.3
		CD	56.0	12.0	32.0
**camSOD**	**Mn**	X-ray	56.1	11.6	32.3
		CD	59.0	14.0	27.0

Supplementary Table 2: X-ray crystallography parameters and statistics.
All parameters from the data collection and refinement of the crystallographic structural models of the *S. aureus* SOD isozymes. All structures were deposited in the protein data bank (PDB).

**Data collection**	**MnSOD**	**camSOD**	**SodM-triple**	**SodA-double**	**SodM-double**
Protein name:	SodA	SodM	SodM (I19F, L159G, F160L)	SodA (G159L, L160F)	SodM (L159G, F160L)
Metal loaded *in vitro*:	**Fe**	**Fe**	**Mn**	**Mn**	**Mn**
Date collected:	02/02/14	07/12/13	24/11/17	20/05/18	20/05/18
X-ray source:	I24	I03	I04	I03	I03
Wavelength (Å):	0.969	0.980	0.979	1.550	0.976
Space group:	$P2_1$	$P6_1$	$P2_12_12_1$	$P2_1$	$P6_1$
Cell dimensions:					
a, *b*, *c* (Å):	47.6, 66.4, 108.3	141.95, 141.95, 46.16	57.98, 58.21, 128.38	52.31, 68.22,57.36	114.21, 144.21, 49.22
α, β, γ (°):	90.0, 98.7, 90.0	90.0, 90.0, 120.0	90.0, 90.0, 90.0	90.0, 100.6, 90.0	90.0, 90.0, 120.0
No. of measured reflections:	118068 (9551)	69671 (7378)	305240 (17616)	220182 (8375)	1033772 (51823)
No. of independent reflections:	33834 (2914)	21099 (2214)	44670 (2415)	36588 (2068)	93785 (4617)
Resolution (Å):	47.01 – 2.20 (2.27 – 2.20)	46.47 – 2.40 (2.49 – 2.40)	43.12 – 1.75 (1.78 – 1.75)	43.46 – 1.80 (1.84 – 1.80)	47.20 – 1.50 (1.53 – 1.50)
$CC_{1/2}$:	0.998 (0.749)	0.993 (0.644)	0.996 (0.822)	0.995 (0.730)	0.999 (0.659)
$I/\sigma I$:	9.2 (1.6)	7.6 (1.6)	9.5 (1.6)	10.9 (2.2)	18.6 (1.9)
Completeness (%):	99.3 (98.5)	99.8 (99.9)	100.0 (99.9)	99.4 (96.4)	100.0 (100.0)
Redundancy:	3.5 (3.3)	3.3 (3.3)	6.8 (7.3)	6.0 (4.0)	11.0 (11.2)
**Refinement**					
R_{work}/ R_{free}	20.07 / 26.74	17.43 / 21.93	18.40 / 21.56	17.08 / 20.68	13.84 / 17.19
No. atoms					
Protein	6282	3243	3252	3198	3250
Ligand/Ions	4	2	2	2	2
Water	126	100	323	133	321
B-factors					
Protein	44.9	43.3	25.6	23.3	30.5
Ligand/Ions	36.0	38.5	16.0	11.2	21.4
Water	38.8	31.3	31.0	24.8	38.8
R.M.S. deviations					
Bond lengths (Å)	0.012	0.012	0.012	0.010	0.011
Bond angles (º)	1.48	1.46	1.40	1.56	1.65
PDB code	**6EX3**	**6EX4**	**6EX5**	**6QV9**	**6QV8**

Supplementary Table 3: Structural comparison of MnSOD and camSOD crystal structures. Each crystal structure determined herein was compared with each other, and with those previously published[2], to quantify the similarity of the enzymes' structures. Structural comparison, using secondary structure matching (SSM) within Coot[33], or using least square comparison (LSQ) within ccp4i[34], is presented as an average root mean squared deviation (RMSD) in Å, with the number of residues/atoms included in the analysis in parentheses.

Protein	PDB code	Core residues' RMSD (Å) by SSM to a monomer of MnSOD	Ligand atoms' RMSD by LSQ to MnSOD
MnSOD	5N56	-	-
Mn-camSOD	5N57	0.743 (198)	0.164 (39)
Fe-MnSOD	6EX3	0.388 (197)	0.139 (39)
Fe-camSOD	6EX4	0.762 (198)	0.205 (39)
Mn-camSOD (L159G)	6EX5	0.551 (197)	0.115 (39)
MnSOD (G159L-L160F)	6QV9	0.500 (197)	0.161 (39)
Mn-camSOD (L159G-F160L)	6QV8	0.602 (196)	0.120 (39)

Supplementary Table 4: Structural comparison of metal-ligand bond lengths.
Bond lengths (Å) between the metal cofactor and each coordinating ligand (amino acid sidechains or solvent) were measured for each determined crystal structure of the wild type and variant *S. aureus* SODs (Supplementary Table 3), both from this study and a previous study[2], using PyMol[6]. Resolution limits of the crystallographic data are also given.

Distance to metal atom, (Å)								
**Coordinating residue/ molecule**	**Atom ID**	**Fe-MnSOD 6EX3**	**Mn-MnSOD 5N56**	**Fe-camSOD 6EX4**	**Mn-camSOD 5N57**	**Mn-camSOD (L159G) 6EX5**	**Mn-MnSOD (G159L-L160F) 6QV9**	**Mn-camSOD (L159G-F160L) 6QV8**
**Resolution (Å)**		**2.20**	**2.07**	**2.40**	**2.30**	**1.75**	**1.80**	**1.50**
**Chain A**								
His27	NE2	2.28	2.36	2.16	2.36	2.18	2.18	2.20
His81	NE2	1.98	2.13	2.12	2.33	2.17	2.12	2.15
His165	NE2	2.11	2.12	2.00	1.97	2.01	2.00	2.05
Asp161	OD2	2.34	2.41	2.23	2.30	2.19	2.12	2.17
H_2O	O	2.13	2.22	2.28	2.21	2.21	2.30	2.21
**Chain B**								
His27	NE2	2.12	2.38	2.04	2.35	2.20	2.09	2.21
His81	NE2	2.00	2.17	2.15	2.33	2.22	2.08	2.19
His165	NE2	2.05	2.06	1.86	1.91	2.03	1.95	2.00
Asp161	OD2	2.35	2.20	2.14	2.34	2.20	2.20	2.20
H_2O	O	2.09	2.22	2.33	2.14	2.26	2.27	2.21
**Chain C**								
His27	NE2	2.23						
His81	NE2	2.18						
His165	NE2	1.95						
Asp161	OD2	2.17						
H_2O	O	2.18						
**Chain D**								
His27	NE2	2.14						
His81	NE2	2.08						
His165	NE2	2.05						
Asp161	OD2	2.17						
H_2O	O	1.98						

Supplementary Table 5 (on next page): Structural comparison of metal-ligand bond angles.
Bond angles (°) between the pairs of coordinating ligands (amino acid sidechains or solvent) and the metal cofactor were measured for each determined crystal structure of the wild type and variant *S. aureus* SODs (Supplementary Table 2), both from this study and a previous study[2], using PyMol[6].

Bond angle, atom - metal - atom, (degrees)			6EX3	5N56	6EX4	5N57	6EX5	6QV9	6QV8
			Chain A						
His27 NE2	M	His81 NE2	90.4	95.1	97.3	91.1	96.8	91.9	92.04
His27 NE2	M	Asp161 OD2	77.7	80.2	84.8	87.4	84.3	87.4	84.3
His27 NE2	M	His165 NE2	89.1	89.6	88.1	89.3	92.9	91.8	91.2
His27 NE2	M	HOH	162.6	167.6	179.6	174.3	169.2	175.5	171.7
His81 NE2	M	Asp161 OD2	113.96	116.5	113.2	120	111.9	111.9	109.7
His81 NE2	M	His165 NE2	133.8	131.1	126.6	124.7	129.6	132.01	130.1
His81 NE2	M	HOH	96.6	95.5	82.4	84.2	89.7	87.9	92.8
His165 NE2	M	HOH	97.4	89.3	92.2	96.1	89.5	91.6	90.9
Asp161 OD2	M	His165 NE2	111.04	112.3	120.1	115.2	118.3	116.05	120.2
Asp161 OD2	M	HOH	84.9	88.9	95.2	92.1	85.3	88.5	87.7
			Chain B						
His27 NE2	M	His81 NE2	94.8	89.8	93.02	91.4	91.7	93.6	92.1
His27 NE2	M	Asp161 OD2	72.3	83.6	93.4	89.4	84.8	88.9	83.3
His27 NE2	M	His165 NE2	87.8	90.3	85.9	87.5	92.3	91.8	92.3
His27 NE2	M	HOH	172.3	171.1	174.1	172.3	172.1	175.56	171.8
His81 NE2	M	Asp161 OD2	112.8	107.6	118.1	122.5	109.4	113.3	110.5
His81 NE2	M	His165 NE2	137.4	133.2	125.5	121.5	131.5	131.01	129.6
His81 NE2	M	HOH	90.5	92.6	90.7	95.7	93.2	81.9	92.2
His165 NE2	M	HOH	92	94.2	95.6	91.3	89.1	87.9	90.4
Asp161 OD2	M	His165 NE2	109.8	118.9	116.4	116	119.1	115.5	119.9
Asp161 OD2	M	HOH	89	87.7	80.7	84.3	87.8	87.2	88.6

			6EX3	
			Chain C	Chain D
His27 NE2	M	His81 NE2	85.6	93.3
His27 NE2	M	Asp161 OD2	84.5	85.8
His27 NE2	M	His165 NE2	95.8	93.3
His27 NE2	M	HOH	170.7	172.4
His81 NE2	M	Asp161 OD2	102.9	117.4
His81 NE2	M	His165 NE2	133.1	138.7
His81 NE2	M	HOH	88.6	91.8
His165 NE2	M	HOH	93.5	86.5
Asp161 OD2	M	His165 NE2	123.9	103.7
Asp161 OD2	M	HOH	89.7	86.9

Supplementary Table 6: Electronic parameters derived from HFEPR spectroscopy.
Parameters (*D*, *E*, and |*D*|+*E*) of the Mn(II) zero-field interaction, calculated from the HFEPR spectra shown in Fig. 3a (*n*=1) using the approach defined in Supplementary Fig. 6 and Supplementary Note 1, with the relevant enzymatic activity with manganese detected and the cambialism ratio observed shown for each isoform.

	D	*E*	|*D*|+*E*	Cambialism Ratio	Mn Activity
MnSOD	-10.86	0.72	11.58	0.002	1836
MnSOD (G159L)	-10.85	0.46	11.31	0.041	942
MnSOD (G159L-L160F)	-10.78	0.47	11.25	0.486	173
MnSOD (F19I-G159L-L160F)	-10.86	0.49	11.36	0.108	526
camSOD	-10.72	0.55	11.27	0.996	251
camSOD (L159G)	-10.72	0.70	11.42	0.030	808
camSOD (L159G-F160L)	-10.90	0.70	11.61	0.016	687
camSOD (I19F-L159G-F160L)	-10.60	0.60	11.20	0.049	789

Supplementary Table 7. Oligonucleotides used for the preparation of mutant variant SODs
Table containing the sequences of all oligonucleotide primers used in this study, with upper case text illustrating site of mutations introduced using QuikChange mutagenesis. All oligonucleotides were purchased from Sigma-Aldrich.

Primer	Sequence	Application
**SDM_SodA19_F**	agaaccacatATTgacaaagaaac	F19I mutant
**SDM_SodA19_R**	gtttctttgtcAATatgtggttct	F19I mutant
**SDM_SodA159/160_F**	cacctattttaCTGTTCgacgtatgggaacacgcttattac	G159L, L160F mutant
**SDM_SodA159/160_R**	gtaataagcgtgttcccatacgtcGAACAGtaaaataggtg	G159L, L160F mutant
**SDM_SodA159_F1**	gggtaaaacacctattttaCTGttagacgtatgggaacacg	G159L mutant
**SDM_SodA159_R1**	cgtgttcccatacgtctaaCAGtaaaataggtgttttaccc	G159L mutant
**SDM_SodA160_F**	cacctattttaggtTTCgacgtatgggaacacg	L160F mutant
**SDM SodA160_R**	cgtgttcccatacgtcGAAacctaaaataggtg	L160F mutant
**SDM_SodM19_F**	ggaaccatatTTCgatcaaagaacaatgg	I19F mutant
**SDM_SodM19_R**	ccattgttctttgatcGAAatatggttcc	I19F mutant
**SDM_SodM159/160_F**	accaatcttaGGCCTGgatgtttgggagcatgcctac	L159G, F160L mutant
**SDM_SodM159/160_R**	gtaggcatgctcccaaacatcCAGGCCtaagattggt	L159G, F160L mutant
**SDM_SodM159_F1**	caccaatcttaGGAttCgatgtttgggagcatgcc	L159G mutant
**SDM_SodM159_R1**	ggcatgctcccaaacatcgaaTCCtaagattggtg	L159G mutant
**SDM_SodM160_F**	caccaatcttactaCTGgatgtttgggagcatgcc	F160L mutant
**SDM_SodM160_R**	ggcatgctcccaaacatcCAGtagtaagattggtg	F160L mutant

Supplementary Note 1: Determination of *D* and *E* values from HFEPR spectra.
The zero-field D and E values were obtained directly from the spectra (Fig. 3a) using the three $D_{nn,-5/2}$ (where nn=xx, yy and zz and -5/2 is the electron spin quantum number, m_s) field positions illustrated in Supplementary Figure 6. These positions are given with respect to $\nu/g\beta$ (where ν is the microwave frequency, g=2.0010 and β=13.996246 GHz T^{-1}). The values could be 'read-off' using the first order equations:

(1) $$D^1_{xx,m_s} = \frac{2\nu-(6m_s+3)E-(2m_s+1)D}{2g\beta}$$

(2) $$D^1_{yy,m_s} = \frac{2\nu+(6m_s+3)E-(2m_s+1)D}{2g\beta}$$

(3) $$D^1_{zz,m_s} = \frac{\nu+(2m_s+1)D}{g\beta}$$

However, since $|D|/\nu$ was large, equations were required for the field positions, to second-order in D and E, to achieve greater accuracy. They were derived from the work of Bir[8]:

(4) $$D^2_{xx,m_s} = \frac{16\nu^2-(12m_s{}^2+12m_s-29)E^2}{16\nu g\beta} - \frac{(24\,\nu(2m_s+1)-2(12m_s{}^2+12m_s-29)D)E}{16\nu g\beta} - \frac{(12m_s{}^2+12m_s-29)D^2-8\nu(2m_s+1)D}{16\nu g\beta}$$

(5) $$D^2_{yy,m_s} = \frac{16\nu^2-(12m_s{}^2+12m_s-29)E^2}{16\nu g\beta} - \frac{(-24\,\nu(2m_s+1)+2(12m_s{}^2+12m_s-29)D)E}{16\nu g\beta} - \frac{(12m_s{}^2+12m_s-29)D^2-8\nu(2m_s+1)D}{16\nu g\beta}$$

(6) $$D^2_{zz,m_s} = \frac{4\nu^2-(12m_s{}^2+12m_s-29)E^2+4\nu(2m_s+1)D}{4\nu g\beta}$$

.
The $D_{zz,-3/2}$ field position was also useful. First, the first-order estimate of *E* was obtained from the difference of the $D_{xx,-5/2}$ and $D_{yy,-5/2}$ field positions. This *E* value was used to estimate *D* to second-order using the difference, $D_{zz,-3/2}$- $D_{zz,-5/2}$, and the corresponding second-order equations. The second-order *D* value was then used to obtain a second-order estimate of *E*. The last two steps were repeated after which the values became self-consistent. The use of differences rather than absolute field positions removed systematic errors arising from the measurement of the magnetic-field.

Supplementary Note 2: Theoretical calculations of the relationship between SOD auto-oxidation and reduction potential.

Assuming the auto-oxidation proceeds by the reverse enzymatic reaction:

(7) $$4O_2 + 4H^+ + 4Mn^{2+}SOD \rightarrow 4HO_2^{\bullet} + 4Mn^{3+}SOD$$

and that the superoxide and resulting peroxide spontaneously disproportionate:

(8) $$4HO_2^{\bullet} \rightarrow 2H_2O_2 + 2O_2$$

(9) $$2H_2O_2 \longrightarrow 2H_2O + O_2$$

The resulting total reaction is:

(10) $$O_2 + 4H^+ + 4Mn^{2+}SOD \longrightarrow 2H_2O + 4Mn^{3+}SOD$$

In redox terms one can express the reaction as:

(11) $$O_2 + 4H^+ + 4e^- \longrightarrow H_2O$$ E^o = 815 mV at pH 7

(12) $$4Mn^{2+}\mathrm{SOD} \longrightarrow 4Mn^{3+}SOD + 4e^-$$

According to the Nernst Equation at equilibrium:

(13) $$E^0 = E^0_{O_2} - E^0_{SOD} = \frac{0.0592}{n} log_{10}K_{eq}$$

and:

(14) $$K_{eq} = \frac{[Mn^{3+}SOD]^4[H_2O]^2}{[Mn^{2+}SOD]^4[O_2][H^+]^4}$$

which reduces to:

(15) $$K_{eq}(aq - pH7) = \frac{[Mn^{3+}SOD]^4}{[Mn^{2+}SOD]^4[O_2]}$$

The oxygen solubility in an air saturated solution at 25°C is about 0.3 mM yielding:

(16) $$E^0_{SOD} = 0.815 - \frac{0.0592}{4} log \frac{MnSOD_{Ox/Red}\, ratio}{0.3 \cdot 10^{-3}}$$

or:

(17) $$E^0_{SOD} = 4.34 - 0.0148\, logMnSOD_{Ox/Red}\, ratio$$

One can consider three limiting cases:

No oxidation	: $MnSOD_{Ox/RedRatio}$= $1x10^{-10}$	or E^o_{SOD} = 0.91 (“wrong metal”)
Half oxidized	: $MnSOD_{Ox/RedRatio}$= 1	or E^o_{SOD} = 0.76 (cambialistic)
Fully auto-oxidized	: $MnSOD_{Ox/RedRatio}$= $1x10^{10}$	or E^o_{SOD} = 0.61 (“right metal”)

For *E. coli* MnSOD E^o has been measured to be 0.29 V, while for Mn(Fe)SOD (the manganese-loaded form of FeSOD), which remains reduced, has been estimated to be greater 0.93. Hence, the auto-oxidation observations are qualitatively consistent with known $E^o_{SOD.}$

Supplementary References

1. Garcia, Y.M. et al. A Superoxide Dismutase Capable of Functioning with Iron or Manganese Promotes the Resistance of *Staphylococcus aureus* to Calprotectin and Nutritional Immunity. *PLoS Pathog* **13**, e1006125 (2017).
2. Barwinska-Sendra, A., Basle, A., Waldron, K.J. & Un, S. A charge polarization model for the metal-specific activity of superoxide dismutases. *Phys Chem Chem Phys* **20**, 2363-2372 (2018).
3. Tarrant, E. et al. Copper stress in Staphylococcus aureus leads to adaptive changes in central carbon metabolism. *Metallomics* **11**, 183-200 (2019).
4. Sievers, F. et al. Fast, scalable generation of high-quality protein multiple sequence alignments using Clustal Omega. *Mol Syst Biol* **7**, 539 (2011).
5. Paxman, J.J. & Heras, B. Bioinformatics Tools and Resources for Analyzing Protein Structures. *Methods Mol Biol* **1549**, 209-220 (2017).
6. Schrodinger, LLC. The PyMOL Molecular Graphics System, Version 1.8. (2015).
7. Tabares, L.C., Gatjens, J. & Un, S. Understanding the influence of the protein environment on the Mn(II) centers in Superoxide Dismutases using High-Field Electron Paramagnetic Resonance. *Biochim Biophys Acta* **1804**, 308-17 (2010).
8. Bir, G.L. Intensity of the allowed and forbidden Electron Paramagnetic Lines. *Sov. Physics-Solid State* **5**, 1628-35 (1964).
9. Fonseca-Junior, N.J., Afonso, M.Q.L., Oliveira, L.C. & Bleicher, L. PFstats: A Network-Based Open Tool for Protein Family Analysis. *J Comput Biol* **25**, 480-486 (2018).
10. Wintjens, R., Gilis, D. & Rooman, M. Mn/Fe superoxide dismutase interaction fingerprints and prediction of oligomerization and metal cofactor from sequence. *Proteins* **70**, 1564-77 (2008).
11. Shannon, P. et al. Cytoscape: a software environment for integrated models of biomolecular interaction networks. *Genome Res* **13**, 2498-504 (2003).
12. Clamp, M., Cuff, J., Searle, S.M. & Barton, G.J. The Jalview Java alignment editor. *Bioinformatics* **20**, 426-7 (2004).
13. Tu, W.Y. et al. Cellular iron distribution in *Bacillus anthracis*. *J Bacteriol* **194**, 932-40 (2012).
14. Li, W. et al. Probing the metal specificity mechanism of superoxide dismutase from human pathogen Clostridium difficile. *Chem Commun (Camb)* **50**, 584-6 (2014).
15. Martin, M.E. et al. A *Streptococcus mutans* superoxide dismutase that is active with either manganese or iron as a cofactor. *J Biol Chem* **261**, 9361-7 (1986).
16. Yamano, S., Sako, Y., Nomura, N. & Maruyama, T. A cambialistic SOD in a strictly aerobic hyperthermophilic archaeon, Aeropyrum pernix. *J Biochem* **126**, 218-25 (1999).
17. Whittaker, M.M. & Whittaker, J.W. Recombinant superoxide dismutase from a hyperthermophilic archaeon, Pyrobaculum aerophilium. *J Biol Inorg Chem* **5**, 402-8 (2000).
18. Amano, A. et al. Characterization of superoxide dismutases purified from either anaerobically maintained or aerated Bacteroides gingivalis. *J Bacteriol* **172**, 1457-63 (1990).
19. Gabbianelli, R. et al. Metal uptake of recombinant cambialistic superoxide dismutase from Propionibacterium shermanii is affected by growth conditions of host Escherichia coli cells. *Biochem Biophys Res Commun* **216**, 841-7 (1995).
20. Meier, B., Barra, D., Bossa, F., Calabrese, L. & Rotilio, G. Synthesis of either Fe- or Mn-superoxide dismutase with an apparently identical protein moiety by an anaerobic bacterium dependent on the metal supplied. *J Biol Chem* **257**, 13977-80 (1982).
21. Tabares, L.C., Bittel, C., Carrillo, N., Bortolotti, A. & Cortez, N. The single superoxide dismutase of Rhodobacter capsulatus is a cambialistic, manganese-containing enzyme. *J Bacteriol* **185**, 3223-7 (2003).
22. Vance, C.K. & Miller, A.F. Novel insights into the basis for *Escherichia coli* superoxide dismutase's metal ion specificity from Mn-substituted FeSOD and its very high E(m). *Biochemistry* **40**, 13079-87 (2001).
23. Gregory, E.M. & Dapper, C.H. Isolation of iron-containing superoxide dismutase from *Bacteroides fragilis*: reconstitution as a Mn-containing enzyme. *Arch Biochem Biophys* **220**, 293-300 (1983).
24. Horsburgh, M.J. et al. MntR modulates expression of the PerR regulon and superoxide resistance in Staphylococcus aureus through control of manganese uptake. *Mol Microbiol* **44**, 1269-86 (2002).

25. Radin, J.N., Zhu, J., Brazel, E.B., McDevitt, C.A. & Kehl-Fie, T.E. Synergy between Nutritional Immunity and Independent Host Defenses Contributes to the Importance of the MntABC Manganese Transporter during Staphylococcus aureus Infection. *Infect Immun* **87**(2019).
26. Handke, L.D., Gribenko, A.V., Timofeyeva, Y., Scully, I.L. & Anderson, A.S. MntC-Dependent Manganese Transport Is Essential for Staphylococcus aureus Oxidative Stress Resistance and Virulence. *mSphere* **3**(2018).
27. Corbin, B.D. et al. Metal chelation and inhibition of bacterial growth in tissue abscesses. *Science* **319**, 962-5 (2008).
28. Choby, J.E. & Skaar, E.P. Heme Synthesis and Acquisition in Bacterial Pathogens. *J Mol Biol* **428**, 3408-28 (2016).
29. Hammer, N.D. & Skaar, E.P. Molecular mechanisms of Staphylococcus aureus iron acquisition. *Annu Rev Microbiol* **65**, 129-47 (2011).
30. Skaar, E.P., Humayun, M., Bae, T., DeBord, K.L. & Schneewind, O. Iron-source preference of Staphylococcus aureus infections. *Science* **305**, 1626-8 (2004).
31. de Beer, T.A., Berka, K., Thornton, J.M. & Laskowski, R.A. PDBsum additions. *Nucleic Acids Res* **42**, D292-6 (2014).
32. Whitmore, L. & Wallace, B.A. DICHROWEB, an online server for protein secondary structure analyses from circular dichroism spectroscopic data. *Nucleic Acids Res* **32**, W668-73 (2004).
33. Emsley, P., Lohkamp, B., Scott, W.G. & Cowtan, K. Features and development of Coot. *Acta Crystallogr D Biol Crystallogr* **66**, 486-501 (2010).
34. Potterton, E., Briggs, P., Turkenburg, M. & Dodson, E. A graphical user interface to the CCP4 program suite. *Acta Crystallogr D Biol Crystallogr* **59**, 1131-7 (2003).
