# Supplementary material for: An evolutionary path to altered cofactor specificity in a metalloenzyme
Source: Nat Commun. 2020 Jun 1;11:2738. doi: 10.1038/s41467-020-16478-0 (PMC7264356; doi:10.1038/s41467-020-16478-0)
Supplement: Supplementary file 3 — Description of Additional Supplementary Files [file 41467_2020_16478_MOESM3_ESM.docx]

**Description of Additional Supplementary Files**

**File Name: Supplementary Data 1**

**Description: SOD sequences and alignment.** An alignment of 2,691 SOD sequences, sampled across the tree of life (SOD_full_alignment_mafft, SOD_trimmed_alignment_mafft_trimal), was used to perform maximum likelihood phylogenetic analysis (SOD_tree_RAXML_WAG.tre). The phylogenetic tree of analysed staphylococcal species (Staphylococcus_species_tree_IQtree_LG_F_R3.tre) was generated based on an alignment of 24,091 concatenated amino acid sites (Staphylococcus_species_tree_alignemnt_mafft_trimal).

**File Name: Supplementary Data 2**

**Description: SOD amino acid correlation analysis.** Dataset is composed of the co-occurring amino acid pairs (residue_pairs_correlation), identified in the global alignment of SODs (Supplementary Data 1). Quantitative output of the amino acid correlation analyses with PFstat is presented (Correlation) ranging from maximum negative correlation (-294) to maximum positive correlation (320). The residue position in the global alignment versus position in *S. aureus* SOD reference sequence is specified (alignment_v_SODseq_position). ID: positions; Residue pair, XY: residue at position; Correlation: negative numbers ascribe anti-correlation.

**File Name: Supplementary Data 3**

**Description: Protein network analyses results.** Protein similarity networks were generated out of 71,757 protein sequences encoded in 29 analyzed staphylococcal genomes using EGN. The dataset consists of groups of protein sequences identified within protein similarity networks, categorised based on the following criteria: The ‘in all’ networks were defined as those networks containing only a single homologue in each of the analyzed genomes. The ‘aureus_argenteus-specific’ networks were defined as networks that contain protein homologues from 7 out of 7 genomes from camSOD-positive species, and none from the camSOD-negative species. The ‘not in aureus_argenteus’ networks were defined as networks that do not contain protein homologues from any of the camSOD-positive genomes and contain homologues from at least 11 out of 22 camSOD-negative species. The ‘enriched_in_aureus_argenteus’ networks define the camSOD-positive species that contain more protein homologues than any of the camSOD-negative species within the network. The virulence factors identified within network analyses (Supplementary Data 5) are indicated in green. Protein sequences were annotated using eggNOG-mapper v1 and AureoWiki. ID: protein identifier; species: strain name; NCBI_name: protein name as in NCBI, with species name in square brackets; Network_ID: identifier of the network; Gene_name: gene name as defined in AureoWiki; EggNOG: protein family as defined in EggNOG; EggNOG_mapping: function/description as in eggNOG; locus tag: as defined in AureoWiki; old locus tag: as defined in AureoWiki; pan gene symbol: as defined in AureoWiki; product, description: functional assignment as defined in AureoWiki; PFAM description: assignment of sequences to protein families based on hidden Markov models (HMMs) as defined in AureoWiki; PSORTb: theoretical (extra)cellular localisation of proteins as defined in AureoWiki; LocateP: theoretical (extra)cellular localisation of proteins as defined in AureoWiki; SignalP: signal peptide prediction and theoretical (extra)cellular localisation of proteins as defined in AureoWiki; transmembrane helices: THHM-based prediction of trans membrane helices as defined in AureoWiki.

**File Name: Supplementary Data 4**

**Description: Protein sequences used to generate species tree.** This dataset is comprised of the single orthologues from 104 ‘Identified in all’ networks (Supplementary Data 3) that were used to generate a species tree of the analysed staphylococci. Protein sequences were annotated using eggNOG-mapper v1, Patric and AureoWiki: ID_of_Saureus_NCTC8325_homologue: NCBI protein identifier in *S. aureus* strain NCTC8325; Name_of_Saureus_NCTC8325_homologue: protein name as defined in NCBI, with species name in square brackets; Network_ID: identifier of the network; Gene_name: gene name as defined in AureoWiki ; pan gene symbol: as defined in AureoWiki; Essential: gene essentiality as defined in Patric and AureoWiki.

**File Name: Supplementary Data 5**

**Description: Genes inferred to be essential and annotated as virulence factors in *S. aureus* NCTC8325 genome.** The virulence_Patric_and_VFDB group (highlighted in green), containing *S. aureus* NCTC8325 virulence factors (as annotated in PATRIC and VFDB databases), and Essential_NCTC_Patric_Aureowiki group (highlighted in blue), containing of *S. aureus* NCTC8325 essential genes (as annotated in PATRIC and AureoWiki databases), are listed alongside genes identified IN_ALL_staphylococci (highlighted in black)**,** Enriched_in_aureus (highlighted in orange) and aureus_specific (highlighted in red) based on protein similarity networks analyses (Supplementary Data 3). The ‘in all’ networks were defined as those networks containing only a single homologue in each of the analyzed genomes, the ‘aureus_specific’ networks were defined as networks containing protein homologues from 7 out of 7 genomes from camSOD-positive species, and none from the camSOD-negative species, the ‘enriched_in_aureus_argenteus’ defines each camSOD-positive species containing more protein homologues than any of the camSOD-negative species within the network. Notes: locus_tag: NCBI *S. aureus* NCTC8325 gene locus; NCBI_ID: protein identifier as in NCBI; Gene_name: gene name as defined in AureoWiki; pan_gene_symbol: as defined in AureoWiki.

**File Name: Supplementary Data 6**

**Description: Known staphylococcal Mn and Fe acquisition systems extracted from protein network analyses.** Protein similarity networks were generated out of 71,757 protein sequences encoded in 29 analyzed Staphylococcal genomes using EGN. Protein sequences were annotated using eggNOG-mapper v1 and AureoWiki. In the heatmap (Supplementary Figure 12 a), homologues identified within a single network of a larger protein family (e.g. network_ID 115, containing IsdF, SirC, SirB, SstA etc.) were identified based on the topology of a phylogenetic tree, inferred from the alignment of all protein sequences found within the network. Notes: ID: unique protein identifier within the network analysis; Network_ID: network identifier; NCBI_ID: protein identifier as defined in NCBI; species: strain name; Gene_name: as defined in AureoWiki; pan_gene_symbol: as defined in AureoWiki; EggNOG: protein family as defined in EggNOG; EggNOG_mapping: protein function/description as defined in EggNOG; product: functional assignment as defined in AureoWiki; description: functional assignment as in AureoWiki; PFAM_description: assignment of sequences to protein families based on HMMs as defined in AureoWiki; effector: biomolecules modulating the activity of regulators, as defined in AureoWiki; PSORTb: theoretical (extra)cellular localisation of proteins as defined in AureoWiki; LocateP: theoretical (extra)cellular localisation of proteins as defined in AureoWik; SignalP: signal peptide prediction and theoretical (extra)cellular localisation of proteins as defined in AureoWiki; transmembrane helices: THHM-based prediction of transmembrane helices as defined in AureoWiki; protein_RefSeq: NCBI protein reference sequence; NCBI_name: protein name as defined in NCBI, with species name in square brackets.
