# Supplementary material for: An evolutionary path to altered cofactor specificity in a metalloenzyme
Source: Nat Commun. 2020 Jun 1;11:2738. doi: 10.1038/s41467-020-16478-0 (PMC7264356; doi:10.1038/s41467-020-16478-0)
Supplement: Supplementary file 4 — Supplementary Data 1 [file 41467_2020_16478_MOESM4_ESM.zip › Supplementary Data 1 Legend.docx]

**Supplementary Data 1: SOD sequences and alignment**

An alignment of 2,691 SOD sequences, sampled across the tree of life (SOD_full_alignment_mafft, SOD_trimmed_alignment_mafft_trimal), was used to perform maximum likelihood phylogenetic analysis (SOD_tree_RAXML_WAG.tre). The phylogenetic tree of analysed staphylococcal species (Staphylococcus_species_tree_IQtree_LG_F_R3.tre) was generated based on an alignment of 24,091 concatenated amino acid sites (Staphylococcus_species_tree_alignemnt_mafft_trimal).
