# Supplementary material for: An evolutionary path to altered cofactor specificity in a metalloenzyme
Source: Nat Commun. 2020 Jun 1;11:2738. doi: 10.1038/s41467-020-16478-0 (PMC7264356; doi:10.1038/s41467-020-16478-0)
Supplement: Supplementary file 10 — Reporting Summary [file 41467_2020_16478_MOESM10_ESM.pdf]

natureresearch

Corresponding author(s): Kevin Waldron

Last updated by author(s): Apr 22, 2020

Reporting Summary

Nature Research wishes to improve the reproducibility of the work that we publish. This form provides structure for consistency and transparency in reporting. For further information on Nature Research policies, see Authors & Referees and the Editorial Policy Checklist .

Statistics

For all statistical analyses, confirm that the following items are present in the figure legend, table legend, main text, or Methods section.

n/a	Confirmed	
☐	☒	The exact sample size (*n*) for each experimental group/condition, given as a discrete number and unit of measurement
☐	☒	A statement on whether measurements were taken from distinct samples or whether the same sample was measured repeatedly
☐	☒	The statistical test(s) used AND whether they are one- or two-sided<br>*Only common tests should be described solely by name; describe more complex techniques in the Methods section.*
☒	☐	A description of all covariates tested
☐	☒	A description of any assumptions or corrections, such as tests of normality and adjustment for multiple comparisons
☐	☒	A full description of the statistical parameters including central tendency (e.g. means) or other basic estimates (e.g. regression coefficient) AND variation (e.g. standard deviation) or associated estimates of uncertainty (e.g. confidence intervals)
☒	☐	For null hypothesis testing, the test statistic (e.g. *F*, *t*, *r*) with confidence intervals, effect sizes, degrees of freedom and *P* value noted<br>*Give P values as exact values whenever suitable.*
☒	☐	For Bayesian analysis, information on the choice of priors and Markov chain Monte Carlo settings
☒	☐	For hierarchical and complex designs, identification of the appropriate level for tests and full reporting of outcomes
☒	☐	Estimates of effect sizes (e.g. Cohen's *d*, Pearson's *r*), indicating how they were calculated

Our web collection on statistics for biologists contains articles on many of the points above.

Software and code

Policy information about availability of computer code

Data collection	Inductively coupled plasma mass spectrometry data was collected using the PlasmaLab software supplied by Thermo with the x-series instrument. Protein and nucleic acid sequences were acquired from the NCBI server.
Data analysis	Circular dichroism data was deconvoluted using the CDSSTR program with reference data set 4 (250-190 nm) and further analyzed using tools on the DichroWeb server (http://dichroweb.cryst.bbk.ac.uk/html/home.shtml). X-ray crystallography data was processed using xia, phased using Phaser, analysed using ConSurf, and images produced using PyMol, with references given within the Methods section of the main text. Sequence analyses and production of bioinformatics figures in the manuscript made use of publicly available tools (Blast, MAFFT, trimAI, PROTTEST, RAXML, GraphPhiAn, PFstats, CYTOSCAPE, EGN, EggNOG-mapper-v1, igraph, dnet, gplots, R, Biopython, ModelFinder, IQ-TREE, FastTree, FigTree, Archaeopteryx, and BRIG) and databases (Patric, VFDB, AureoWiki), with all citations and links given in the Methods of the manuscript.

For manuscripts utilizing custom algorithms or software that are central to the research but not yet described in published literature, software must be made available to editors/reviewers. We strongly encourage code deposition in a community repository (e.g. GitHub). See the Nature Research guidelines for submitting code & software for further information.

Data

Policy information about availability of data

All manuscripts must include a data availability statement. This statement should provide the following information, where applicable:

- Accession codes, unique identifiers, or web links for publicly available datasets
- A list of figures that have associated raw data
- A description of any restrictions on data availability

All bioinformatic analyses are based on datasets acquired from the NCBI public sequence database, and the sequences used are included in this published article and its supplementary dataset files. Analyses of these sequences exploited public databases: PATRIC v3.6.3 (ftp://ftp.patricbrc.org/specialty_genes/); VFDB (http://www.mgc.ac.cn/cgi-bin/VFs/genus.cgi?Genus=Staphylococcus); and AureoWiki (https://aureowiki.med.uni-greifswald.de/download_gene_specific_information). Protein structural data that support the findings in this study have all been deposited in the Protein Data Bank with accession codes

6EX3, 6EX4, 6EX5, 6QV8 and 6QV9 (available from www.rcsb.org), with these details given in the manuscript text and supplementary information files. Source Data are provided for Figure 6 and Table 1 of the main text and for Supplementary Figure 1 in the SI.

Field-specific reporting

Please select the one below that is the best fit for your research. If you are not sure, read the appropriate sections before making your selection.

- [x] Life sciences
- [ ] Behavioural & social sciences
- [ ] Ecological, evolutionary & environmental sciences

For a reference copy of the document with all sections, see nature.com/documents/nr-reporting-summary-flat.pdf

Life sciences study design

All studies must disclose on these points even when the disclosure is negative.

Sample size	No sample size calculations were performed for this study. Instead, sample sizes were determined from the minimum replicates needed to perform the relevant statistical analyses while balancing the need for production of the biological materials, as usual for biochemistry studies. For biophysical analyses by EPR spectroscopy, a single replicate was used due to the expense and time incurred of repeating these analyses. For CD spectroscopy, a single biological replicate was deemed appropriate as these spectra were analyzed qualitatively and compared with more accurate structural analyses, i.e. X-ray crystallography. We selected n=3 replicates as a minimum for all assays that would be analyzed quantitatively in order that SD or SEM could be calculated and appropriate statistical significance tests applied.
Data exclusions	No data were excluded.
Replication	All sections in which we describe using 'independent biological replicates' involved preparing entirely separate samples using different cell or protein preparations on different days but using identical protocols. All replicates performed were successful and were included in the analysis herein. For biophysical analyses such as EPR and CD spectroscopy, a single biological replicate was deemed appropriate as they were for qualitative analysis only and were always paired with complementary analyses for interpretation (UV/visible spectroscopy and X-ray crystallography, respectively).
Randomization	Randomization was not relevant to the experiments in this study as it involved use of purified proteins and microbial strains rather than patient groups.
Blinding	Randomization was not relevant to the experiments in this study as it involved use of purified proteins and microbial strains rather than patient groups.

Reporting for specific materials, systems and methods

We require information from authors about some types of materials, experimental systems and methods used in many studies. Here, indicate whether each material, system or method listed is relevant to your study. If you are not sure if a list item applies to your research, read the appropriate section before selecting a response.

Materials & experimental systems

n/a	Involved in the study
[x]	[ ] Antibodies
[x]	[ ] Eukaryotic cell lines
[x]	[ ] Palaeontology
[x]	[ ] Animals and other organisms
[x]	[ ] Human research participants
[x]	[ ] Clinical data

Methods

n/a	Involved in the study
[x]	[ ] ChIP-seq
[x]	[ ] Flow cytometry
[x]	[ ] MRI-based neuroimaging
